# The association of vancomycin trough levels with outcomes among patients with methicillin-resistant *Staphylococcus aureus* (MRSA) infections: Retrospective cohort study

**DOI:** 10.1371/journal.pone.0214309

**Published:** 2019-04-04

**Authors:** Dafna Yahav, Maria Abbas, Laila Nassar, Alia Ghrayeb, Daniel Kurnik, Daniel Shepshelovich, Leonard Leibovici, Mical Paul

**Affiliations:** 1 Infectious Diseases Unit, Rabin Medical Center, Beilinson Hospital, Petah Tikva, Israel; 2 Sackler School of Medicine, Tel Aviv University, Ramat Aviv, Israel; 3 Rappaport Faculty of Medicine, Technion-Israel Institute of Technology, Haifa, Israel; 4 Clinical Pharmacology Unit, Rambam Health Care Campus, Haifa, Israel; 5 Medicine A, Rabin Medical Center, Beilinson Hospital, Petah Tikva, Israel; 6 Medicine E, Rabin Medical Center, Beilinson Hospital, Petah Tikva, Israel; 7 Institute of Infectious Diseases, Rambam Health Care Campus, Haifa, Israel; University Medical Center Groningen, NETHERLANDS

## Abstract

**Introduction:**

Current guidelines recommend maintaining vancomycin trough concentrations between 15–20 mg/L for serious methicillin resistant *staphylococcus aureus* (MRSA) infections. This recommendation is based on limited evidence.

**Methods:**

A retrospective study including patients with vancomycin susceptible MRSA infections (MIC< = 2 mg/L), treated with vancomycin. We compared outcomes among patients attaining high (> = 15mg/L) vs low (<15mg/L) trough vancomycin levels. We used a propensity score to matching patients achieving low and high levels and conducted an adjusted analysis in the propensity score (PS)-matched cohort using regression analysis. Primary outcome was 30-day all-cause mortality.

**Results:**

Among 285 patients included, there were no significant differences between patients achieving high and low vancomycin levels in mortality (46/131, 35.1% vs 41/154, 26.6%), clinical success, microbiological success, or nephrotoxicity. Similarly, in the PS-matched cohort (n = 162), there was no significant difference in mortality between patients with high and low vancomycin levels (24/53, 45.3% vs 57/109, 52.3%, respectively), adjusted odds ratio for mortality with high levels 0.63 (95% confidence interval 0.28–1.43). In both cohorts, patients with pneumonia achieving high levels had significantly higher clinical and microbiological success (PS-matched cohort: clinical success: 16/32, 50.0% vs 5/27, 18.5%, p = 0.012; microbiological success: 19/32, 59.4% vs 7/27, 25.9%, p = 0.010), without significant differences in mortality.

**Conclusions:**

We found no association between vancomycin levels > = 15 mg/L and clinical outcomes in patients with MRSA infections. In patients with MRSA pneumonia, vancomycin levels > = 15 mg/L were associated with higher clinical success rates. Further larger cohort studies are needed to define optimal vancomycin levels according to the site of infection.

## Introduction

The Infectious Diseases Society of America (IDSA) guidelines for the treatment of methicillin resistant *Staphylococcus aureus* (MRSA) infections recommend maintaining vancomycin trough concentrations of 15–20 mg/L for serious infections. The rational for achieving higher levels is to improve pharmacodynamics and minimize selection of resistant strains. [1] These recommendations address severe MRSA infections in general, without differentiating patients by the site of infection.

Current data on the effectiveness of higher vancomycin trough levels in the treatment of MRSA infections are limited to few prospective and mainly small retrospective studies. In a recent systematic review and meta-analysis of non-randomized studies, higher trough levels were associated with microbiological and clinical success in various MRSA infections, but not with lower mortality. However, in studies including predominantly patients with pneumonia, lower mortality was found in the group with vancomycin levels > = 15 mg/L. [2] Another recent systematic review and meta-analysis found no significant difference in mortality or clinical success. [3] In both meta-analyses, higher rates of nephrotoxicity were observed with higher trough levels. [2,3]

We conducted a retrospective study evaluating the effectiveness and safety of high vs. low vancomycin trough levels in patients with serious MRSA infections, overall and separately according to different sites of infection, with special emphasis on pneumonia; and used a propensity score to minimize bias.

## Materials and methods

### Study design and population

This was a retrospective cohort study, conducted in two tertiary care university hospitals in Israel. The study was approved by the local ethical committees of both Rabin Medical Center and Rambam Medical Center. Due to the retrospective design informed consent was waived by the ethical committees. We included adult patients hospitalized between January 1, 2013 and December 31, 2015 who were treated with vancomycin for a documented infection caused by MRSA susceptible to vancomycin (MIC< = 2), and in whom vancomycin levels were measured at least once. Documented infection was defined as any infection fulfilling CDC/NHSN surveillance definition of health care-associated infection, [4] with isolation of MRSA from the site of infection or blood. Patients were identified using the pharmacology and microbiology laboratory computerized databases; inclusion criteria were applied to all patients with at least one vancomycin level measurement and a concurrent MRSA isolate. Each patient was included once (first episode). Polymicrobial infections were excluded. We also excluded patients hospitalized in the intensive care unit (ICU), a unique group in terms of vancomycin administration, with high rates of sub-therapeutic levels. [5]

Exposure variable: First trough vancomycin level obtained between days 3 to 7 of treatment, dichotomized to high level (> = 15mg/dL) and low level (<15mg/dL). In both hospitals, vancomycin dosing guidelines throughout the study period recommended initial dosing of 1gr every 12 h in adult patients with normal renal function with subsequent dose adjustment according to serum trough levels. Loading doses were not recommended. The recommended target trough levels were 10–20 mg/L. As this was a retrospective study, vancomycin dosage and drug levels testing were determined at the discretion of treating physicians. We documented all vancomycin trough levels available during the treatment course of each patient.

Outcomes: The primary outcome was all-cause 30-day mortality. Secondary outcomes included:

- Clinical success, determined on day 7 following the first positive culture if the patient was afebrile for at least 48 hours (temperature < 38°); hemodynamically stable without need for vasopressors; had pulse < 100 per minute; and had leukocyte count above 4000 and below 12,000 cells/mm^3^ or in accordance to baseline.
- Microbiological success, defined as negative cultures within 7 treatment days according to the source of infection: blood cultures for bacteremia, endocarditis and osteomyelitis; blood and CSF cultures for meningitis; blood and respiratory cultures for pneumonia; blood and tissue cultures for skin and soft tissue infections.
- Nephrotoxicity. In order to define deterioration of renal function we extracted creatinine baseline levels at the start of vancomycin treatment and also maximum creatinine levels from start of vancomycin and up until 4 days after stopping the drug. These data were summarized according to RIFLE criteria comparing start and maximum creatinine. We defined nephrotoxicity as RIFLE of at least 2.

Confounders and other risk factors: We collected patient and infection characteristics for the comparison between the high and low-level patient groups and as confounders for the analysis of mortality. The main confounder assessed was the isolates’ MICs to vancomycin. Other variables included age, gender, functional capacity, Charlson comorbidity index, [6] creatinine and albumin at baseline and PITT bacteremia score as a measure of severity of infection. [7]

#### Definitions

Patients were defined to have pneumonia if they had pneumonia according to CDC criteria for healthcare associated infections with a monomicrobial culture of MRSA from respiratory samples, with no evidence of other infection. [4]

#### Data sources and collection

Data were collected retrospectively by reviewing full electronic patient files in both hospitals. Data on mortality were available from the national registry. Vancomycin MIC determination was performed using Etest (bioMérieux, Marcy l’Etoile, France). Vancomycin levels were determined using chemiluminescent microparticles immunoassay (CMIA Architect i1000).

### Statistical analysis

We compared baseline characteristics and outcomes between the high and low vancomycin level groups. We generated a propensity score (PS) predicting the propensity to achieve high vancomycin levels and created a cohort of patients matched on the PS (PS-matched cohort). Tolerance used for propensity matching was 0.1. We compared outcomes in the PS-matched cohort. Since PS matching did not result in fully comparable patient groups, we performed a multivariable analysis for mortality in the PS-matched cohort, using multiple imputations to complete missing data for the variables included in the regression analysis. In sensitivity analyses, we assessed vancomycin level/MIC and AUC/MIC ratios in place of the dichotomous high vs. low vancomycin levels, in the PS-matched cohort. [8] Proportions were compared using the chi-square test, and continuous variables were compared using the Mann–Whitney test or unpaired Student t-test (for non-normal and normal distributed variables, respectively). Multivariable analyses for the PS and mortality were conducted using logistic regression including variables significant in univariate analyses (P<0.05) and deemed clinically significant. For the multivariate analysis, the Charlson comorbidity index and PITT scores were dichotomized. Dichotomization was performed to maximize the association of the variable with mortality. All analyses were performed using IBM SPSS Statistics 24.

## Results

Overall, 285 patients were included in the study. The mean age was 67 years (standard deviation (SD), 15.8), and 177 (62.1%) were males. Vancomycin levels were collected at least once for all patients included (the median number of measurements of vancomycin concentrations was 5 per patients; interquartile range, 2–5). The mean first vancomycin trough levels were 16.2±9.7 mg/L, median 13.9 (interquartile range, 9.1–21.3). The most common sites of infection were pneumonia (93/285, 33%), skin and soft tissue infections (55/285, 19%), catheter related bloodstream infections (44/285, 15%), bone and joint infections (27/285, 9%) and primary bacteremia (31/285, 11%). Any bacteremia (primary or secondary) was documented in 161 patients (56%).

Baseline characteristics and outcomes of patients with low (<15 mg/L) versus high (> = 15 mg/L) first vancomycin levels are presented in Table 1. Patients achieving higher vancomycin first trough levels had a non-significant trend to higher 30-day mortality, without significant differences in clinical success, microbiological success, or adverse events (including nephrotoxicity). However, patients with higher vancomycin levels were older, more likely to be female, had higher comorbidity score and more severe presentation of infection compared to those with lower vancomycin trough levels (Table 1).

**Table 1:** Characteristics of patients with low versus high vancomycin levels in the entire cohort.

Variable	Vancomycin levels < 15 (154 patients)	Vancomycin levels > = 15 (131 patients)	p-value
Age	63.5 (54.0–77.0)	71.0 (63.0–81.0)	**0.004**
Gender male	114/154 (74.0%)	63/131 (48.1%)	**<0.001**
Independent functional capacity	63/154 (40.9%)	33/131 (25.2%)	**0.005**
Charlson comorbidity index	2.0 (0–3.0)	2.0 (1.0–4.0	0.060
Source bacteremia	89/154 (57.8%)	72/131 (55.0%)	0.631
Pneumonia	43/154 (28.9%)	50/131 (39.1%)	0.076
PITT bacteremia score	1.0 (0–5.0)	4.0 (0–7.0)	**0.025**
Creatinine at infection onset (mg/dL)	1.4 (0.7–2.5) (152 patients)	1.2 (0,9–2.0) (131 patients)	0.472
Albumin at infection onset (gr/dL)	2.5 (2.1–3.1) (117 patients)	2.1 (1.7–2.7) (109 patients)	**<0.001**
Vancomycin MIC > = 1	85/143 (59.4%)	79/127 (62.2%)	0.642
Vancomycin MIC > = 1.5	29/143 (20.3%)	27/127 (21.3%)	0.843
Vancomycin first level (mg/L)	9.6 (7.8–11.9)	22.3 (18.6–26.5)	<0.01
Vancomycin mean level (mg/L)	14.0 (10.3–17.9)	20.6 (18.1–25.0)	<0.01
Use of concomitant nephrotoxic drugs	105/154 (68.2%)	101/131 (77.1%)	0.094
Duration of vancomycin treatment (days)	10 (7–16.8)	12 (7–18)	0.146
Mortality 30 days	41/154 (26.6%)	46/131(35.1%)	0.121
Clinical success	67/153 (43.8%)	55/128 (43.0%)	0.890
Microbiological success	86/151 (57.0%)	69/128 (53.9%)	0.610
RIFLE > = 2 during treatment compared to baseline	38/154 (24.7%)	37/131 (28.2%)	0.495

Categorical variables are given in number (%); continuous variables in median (interquartile range)

Second and third vancomycin levels were both significantly higher in the high-level group and are presented in Figs A and B in S1 File.

Separate analysis of 93 patients with pneumonia demonstrated no significant difference in 30-day mortality between patients with different vancomycin levels in spite of significantly higher clinical and microbiological success rates in the group achieving high vancomycin levels (Table 2).

**Table 2:** Outcomes among patients with pneumonia according to vancomycin levels.

Outcome	Vancomycin levels < 15	Vancomycin levels > = 15	p-value
**Entire cohort (93 patients**)	**43 patients**	**50 patients**	
30 day mortality	17 (39.5%)	21 (42.0%)	0.809
Clinical success	8 (18.6%)	20 (40.0%)	**0.021**
Microbiological success	13 (30.2%)	26 (52.0%)	**0.034**
**PS-matched (59 patients)**	**27 patients**	**32 patients**	
30 day mortality	13 (48.1%)	12 (37.5%)	0.410
Clinical success	5 (18.5%)	16 (50/0%)	**0.012**
Microbiological success	7 (25.9%)	19 (59.4%)	**0.010**

The PS-matched cohort included 162 patients, 81 with vancomycin levels > = 15 mg/L and 81 with levels < 15 mg/L. There were no significant differences in patient characteristics between the low- and high-vancomycin level groups (S1 Table). MIC distribution among the two groups was similar (MIC< = 1: 55/78, 71% in the high-level group, 59/78, 76% in the low-level group). Univariate analysis of risk factors for mortality among these 162 patients is presented in Table 3. Vancomycin levels in this cohort were not associated with 30-day mortality (57/109, 52.3% of surviving patients had high vancomycin trough levels versus 24/53, 45.3% of patients who died, p = 0.402). Analysis of vancomycin levels/ MIC ratio also did not demonstrate a significant association with 30-day mortality (Table 3).

**Table 3:** Univariate analysis of risk factors for mortality among 162 propensity score-matched patients.

Variable	Survived at 30 day (109 patients)	Dead at 30 day (53 patients)	p-value
Age	71.0 (58.5–78.0)	74.0 (66.5–80.0)	**0.051**
Gender male	67/109 (61.5%)	34/53 (64.2%)	0.741
Independent functional capacity	32/109 (29.4%)	14/53 (26.4%)	0.697
Charlson comorbidity index	2.0 (0–3.0)	3.0 (2.0–4.0)	**0.006**
Source bacteremia	61/109 (56.0%)	32/53 (60.4%)	0.594
Pneumonia	34/109 (31.2%)	25/53 (47.2%)	**0.047**
PITT bacteremia score	1.0 (0–5.0)	7.0 (3.5–10.0)	**<0.001**
Creatinine at infection onset (mg/dL)	1.2 (0.7–2.1)	1.8 (1.3–2.9)	**<0.001**
Albumin at infection onset (gr/dL)	2.6 (2.1–3.0)	2.3 (1.7–2.5)	**0.001**
Vancomycin levels > = 15	57/109 (52.3%)	24/53 (45.3%)	0.402
Vancomycin levels to MIC ratio	16.7 (9.8–25.3) (105 patients)	20.9 (12.9–27.9) (48 patients)	0.223
Vancomycin MIC> = 1	69/105 (65.7%)	25/48 (52.1%)	0.108
Vancomycin MIC> = 1.5	28/105 (26.7%)	8/48 (16.7%)	0.176

Categorical variables are given in number (%); continuous variables in median (interquartile range)

Significant risk factors for 30-day mortality on multivariate analysis were age older than 65 years, higher PITT bacteremia score and higher Charlson comorbidity index. Vancomycin levels were not associated with 30-day mortality in multivariate analysis (Table 4). A repeated analysis introducing vancomycin levels to MIC ratio instead of vancomycin levels demonstrated similar results. Calculation of AUC/MIC was possible for 139 patients. No significant difference in 30-day mortality was demonstrated among patients with AUC/MIC> = 400 vs those with lower AUC/MIC in univariate analysis.

**Table 4:** Multivariate analysis of risk factors for mortality among 162 PS-matched patients.

variable	Odds ratio	95% confidence interval	p-value
Vancomycin levels > = 15	0.671	0.304–1.483	0.324
Creatinine at onset (mg/dL)	1.134	0.894–1.440	0.300
Albumin at onset (g/dL)	0.537	0.262–1.099	0.089
Charlson comorbidity index > 3	3.483	1.417–8.559	**0.007**
PITT bacteremia score > 4	6.705	2.694–16.688	**<0.001**
Age	1.041	1.006–1.077	**0.019**
Source pneumonia	0.981	0.409–2.353	0.965

OR > 1 are associated with mortality

In a subgroup of 59 patients with MRSA pneumonia included in the propensity score cohort, we found similar findings as in the entire cohort, i.e. no significant difference in 30-day mortality. However, significantly higher rates of clinical and microbiological success among patients achieving high levels were observed (Table 2).

## Discussion

In a cohort of 285 patients treated with vancomycin for severe MRSA infections, we found no difference in mortality rates between patients achieving recommended vancomycin levels of > = 15 mg/L vs. lower levels. Among 162 propensity score-matched patients, independent risk factors for 30-day mortality were older age, higher PITT bacteremia score and higher Charlson comorbidity index. High vancomycin trough levels were not significantly associated with mortality on univariate or multivariate analysis. Vancomycin levels were also not associated with clinical or microbiological success rates or nephrotoxicity. In a subgroup of patients with pneumonia, we found significantly higher clinical and microbiological success rates with high vancomycin levels, without a significant difference in mortality.

Our results are largely compatible with findings from the previous systematic reviews, failing to identify a clinical benefit for higher vancomycin levels with respect to mortality and clinical cure. [2,3] We did not observe differences in microbiologic failure rates, unlike the finding of significantly higher failure rates in patients with low vancomycin levels in a previous systematic review. [2] Microbiological failure rates were higher in our study than in previous studies, with overall microbiological failure rates of 31% (226/719) in previous studies [2] and 43% (124/285) in ours. Nephrotoxicity was significantly higher with vancomycin levels > = 15 mg/L when previous studies were pooled [2], unlike in our study. Event rates of nephrotoxicity were higher in our study (75/285, 26.3%) compared to the pooled total from Steinmetz et al. meta-analysis [2] (99/607, 16.3%), reflecting different definitions or differences in the patient case-mix.

According to results of our and previous studies, higher vancomycin levels might benefit patients with MRSA pneumonia. We demonstrated higher rates of clinical and microbiological success with high vancomycin trough levels in the subgroup of patients with MRSA pneumonia, with no difference in mortality, while in the above-mentioned systematic review, mortality was significantly lower with higher levels. [2] Vancomycin has lower tissue penetration and achieves lower alveolar lining fluid concentration compared to other anti MRSA drugs. Thus, higher serum concentrations may be necessary in order to achieve appropriate lung concentrations. [9,10]

Our study is one of the largest studies evaluating the association between vancomycin serum concentrations and outcomes of patients with MRSA infections. We restricted inclusion to monomicrobial MRSA infections, and patients included had severe infections, as reflected by the 30-day mortality rates above 30% both overall and in the PS-matched cohort. Limitations of our study are its retrospective design and the fact that it was conducted in Israeli centers, reflecting the local policies of vancomycin use and dosage and therapeutic drug monitoring. In addition, ICU patients were excluded, and thus, conclusions on patients with the most severe infections cannot be drawn from this study. Baseline differences were minimized in the propensity score analysis, nevertheless, we cannot exclude the possibility that residual bias affected our findings.

In conclusion, we found no association between the target vancomycin levels recommended by IDSA guidelines and clinical outcomes in patients with various MRSA infections. In a subgroup of patients with MRSA pneumonia, vancomycin levels > = 15 mg/L were associated with higher rates of clinical success. Further larger cohort studies are needed in order to define optimal vancomycin trough levels in MRSA infections according isolates’ MIC and the site of infection.

## Supporting information

S1 Table (DOCX)

S1 File (DOCX)
