# Supplementary material for: The association of vancomycin trough levels with outcomes among patients with methicillin-resistant Staphylococcus aureus (MRSA) infections: Retrospective cohort study
Source: PLoS One. 2019 Apr 4;14(4):e0214309. doi: 10.1371/journal.pone.0214309 (PMC6448937; doi:10.1371/journal.pone.0214309)
Supplement: S1 Table — (DOCX) [file pone.0214309.s001.docx]

**Supplemental Table 1: Characteristics of patients with low versus high vancomycin levels in the propensity matched cohort**

| **Variable** | **Initial Vancomycin levels < 15 mg/L**  (n=81) | **Initial Vancomycin levels >=15 mg/L** (n=81) | **P-value** |
| --- | --- | --- | --- |
| Age | 73 (65.5-79) | 70 (61-80) | 0.380 |
| Gender male | 54 (66.7) | 47 (58) | 0.256 |
| Independent functional capacity | 23 (28.4) | 23 (28.4) | 1 |
| Charlson comorbidity index | 2 (1-4) | 2 (1-3) | 0.850 |
| Source bacteremia  Pneumonia | 48 (59.3)  27 (33.3) | 45 (55.6)  32 (39.5) | 0.634  0.414 |
| PITT bacteremia score | 2 (1-7.5) | 3 (0-7) | 0.981 |
| Creatinine at infection onset (mg/dL) | 1.5 (0.9-2.7) | 1.3 (0.8-2.3) | 0.293 |
| Albumin at infection onset (gr/dL) | 2.5 (2-3) | 2.4 (2-2.9) | 0.362 |
| Vancomycin MIC >=1 | 43/75 (57.3) | 51/78 (65.4) | 0.306 |
| Vancomycin MIC >=1.5 | 14/75 (18.7) | 22/78 (28.2) | 0.164 |
| Vancomycin first level (mg/L) | 10.1 (8-12) | 22.3 (18.9-28.5) | <0.01 |
| Vancomycin mean level (mg/L) | 15.9 (12.2-18.7) | 20.5 (18.1-25) | <0.01 |
| Use of concomitant nephrotoxic drugs | 59 (72.8) | 59 (72.8) | 1 |
| Duration of vancomycin treatment (days) | 11 (7-16) | 13 (8-21) | 0.169 |
| Mortality at 30 days | 29 (35.8) | 24 (29.6) | 0.402 |
| Clinical success | 32/81 (39.5) | 37/80 (46.3) | 0.387 |
| Microbiological success | 42/78 (53.8) | 46/79 (58.2) | 0.580 |
| RIFLE >=2 during treatment compared to baseline | 23 (28.4) | 27 (33.3) | 0.496 |

Categorical variables are given in number (%); continuous variables in median (interquartile range)
