# Supplementary material for: The association of vancomycin trough levels with outcomes among patients with methicillin-resistant Staphylococcus aureus (MRSA) infections: Retrospective cohort study
Source: PLoS One. 2019 Apr 4;14(4):e0214309. doi: 10.1371/journal.pone.0214309 (PMC6448937; doi:10.1371/journal.pone.0214309)
Supplement: S1 File — (DOCX) [file pone.0214309.s002.docx]

**Supplemental figure 1: Vancomycin second level between low and high levels group:**


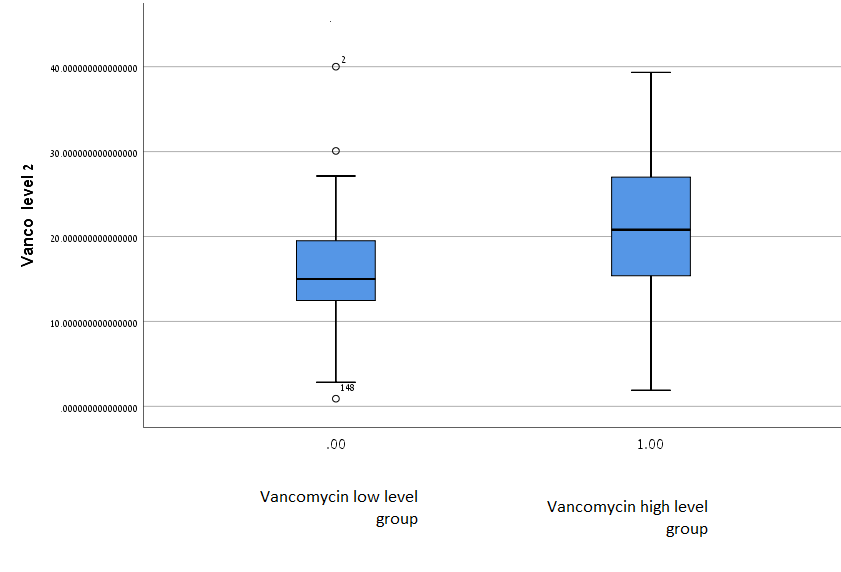


Among 121 patients in the low-level group and 108 patients in the high-level group for whom second vancomycin trough levels were available, median levels were 15.0 (12.4-19.5) and 20.8 (15.3-27.0), respectively, p<0.01.

**Supplemental figure 2: Vancomycin third level between low and high levels group:**


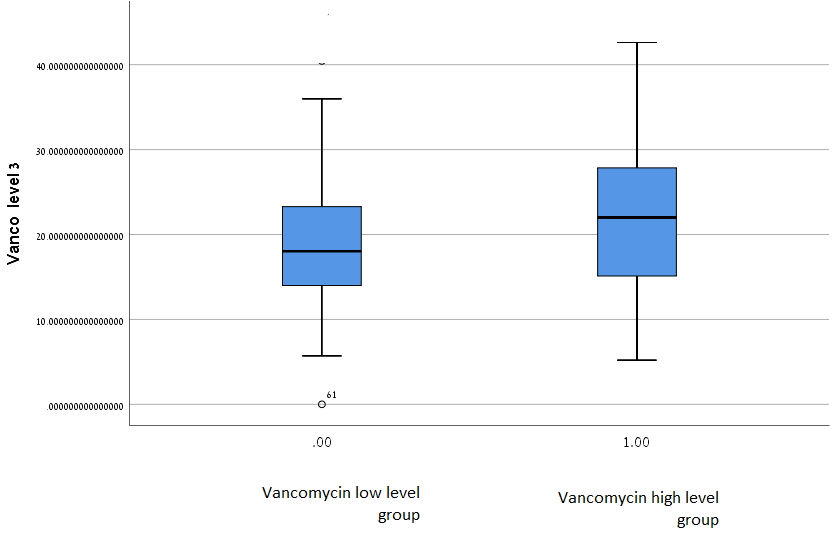


Among 94 patients in the low-level group and 79 patients in the high-level group for whom third vancomycin trough levels were available, median levels were 18.0 (13.9-23.4) and 22.0 (15-27.8), respectively, p=0.05.
